# Clinical characteristics and disease outcomes in non-diabetic chronic kidney disease: retrospective analysis of a US healthcare claims database

**DOI:** 10.1007/s40620-022-01340-x

**Published:** 2022-05-14

**Authors:** Christoph Wanner, Johannes Schuchhardt, Chris Bauer, Stefanie Lindemann, Meike Brinker, Sheldon X. Kong, Frank Kleinjung, Andrea Horvat-Broecker, Tatsiana Vaitsiakhovich

**Affiliations:** 1 Medizinische Klinik und Poliklinik 1, Schwerpunkt Nephrologie, Universitätsklinik Würzburg, Würzburg, Germany; 2 MicroDiscovery GmbH, Berlin, Germany; 3 Bayer AG, Müllerstraße 178, 13353 Berlin, Germany; 4 Bayer AG, Wuppertal, Germany; 5 Bayer Pharmaceuticals, Whippany, NJ USA

**Keywords:** Chronic kidney disease, Non-diabetic CKD, Real-world evidence, Claims database

## Abstract

**Background:**

The observational, real-world evidence FLIEDER study aimed to describe patient clinical characteristics and investigate clinical outcomes in non-diabetic patients with chronic kidney disease (CKD) using data collected from routine clinical practice in the United States.

**Methods:**

Between 1 January, 2008–31 December, 2018, individuals aged ≥ 18 years, with non-diabetic, stage 3–4 CKD were indexed in the Optum^®^ Clinformatics^®^ Data Mart US healthcare claims database using International Classification of Diseases-9/10 codes for CKD or by laboratory values (estimated glomerular filtration rate [eGFR] 15–59 mL/min/1.73 m^2^). The primary outcomes were hospitalization for heart failure, a composite kidney outcome of end-stage kidney disease/kidney failure/need for dialysis and worsening of CKD stage from baseline. The effects of the intercurrent events of a sustained post-baseline decline in eGFR ≥ 30%, ≥ 40%, and ≥ 57% on the subsequent risk of the primary outcomes were also assessed.

**Results:**

In the main study cohort (*N* = 504,924), median age was 75.0 years, and 60.5% were female. Most patients (94.7%) had stage 3 CKD at index. Incidence rates for hospitalization for heart failure, the composite kidney outcome, and worsening of CKD stage from baseline were 4.0, 10.3, and 4.4 events/100 patient-years, respectively. The intercurrent event analysis demonstrated that a relative decline in kidney function from baseline significantly increased the risk of cardiorenal events.

**Conclusions:**

This real-world study highlights that patients with non-diabetic CKD are at high risk of serious adverse clinical outcomes, and that this risk is amplified in patients who experienced greater post-baseline eGFR decline.

**Graphical abstract:**

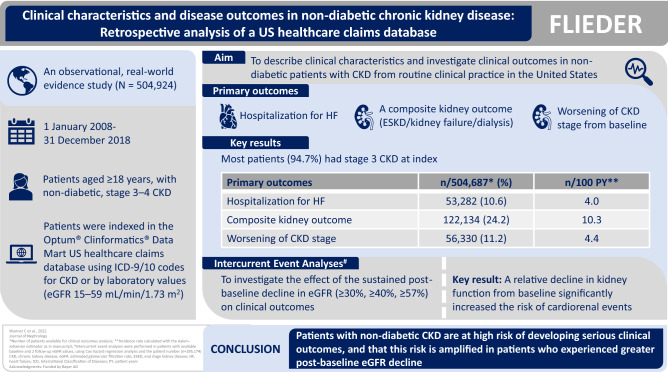

**Supplementary Information:**

The online version contains supplementary material available at 10.1007/s40620-022-01340-x.

## Introduction

By 2040, chronic kidney disease (CKD) is expected to be the fifth leading cause of years of life lost worldwide, advancing from sixteenth place in 2016 [1]. Although diabetes is well recognized as a leading cause of CKD globally [2, 3], a substantial proportion of global CKD burden is non-diabetic in origin and attributed to other causes, such as hypertension [2, 4].

People with CKD are 5–10 times more likely to die prematurely than progress to end-stage kidney disease (ESKD), with the risk of death rising exponentially as kidney function worsens[2, 5]. In addition, cardiovascular (CV) mortality presents a competing risk for patients with CKD [6]. A meta-analysis of > 1.5 million individuals from general, high-risk, and CKD populations showed that decreasing glomerular filtration rate and increasing albuminuria were associated with increases in all-cause mortality, CV mortality, acute kidney injury (AKI) incidence, and kidney disease progression [7].

Existing therapies that are proven to delay CKD progression to ESKD include angiotensin-converting enzyme inhibitors (ACEis) and angiotensin receptor blockers (ARBs), and newer therapies, such as blood-glucose lowering sodium-glucose co-transporter-2 inhibitors and, most recently, the selective, nonsteroidal mineralocorticoid receptor antagonist finerenone (which does not affect glycated hemoglobin levels). However, most of the data supporting the use of these agents to improve cardiorenal outcomes are from clinical trials in patients with CKD and diabetes [8–13]. Patients with non-diabetic CKD are relatively understudied, and there is a need for improved understanding of the risk factors and outcomes in these patients, and for new therapies to reduce their cardiorenal risk. To address this, we conducted the Exploratory analysis o*F* *L*ong*I*tudinal patient-level Data for non-diab*E*tic ch*R*onic kidney disease in a US claims database (FLIEDER) study to describe clinical characteristics and investigate clinical outcomes in patients from routine clinical practice in the United States.

## Methods

### Study design

Individuals in the Optum^®^ Clinformatics^®^ Data Mart with moderate-to-severe CKD (CKD stage 3–4) and no diagnosis of diabetes were identified between 1 January, 2008 and 31 December, 2018, which was the most recent data cut available during the conduct of the study. The Optum^®^ Clinformatics^®^ Data Mart is a de-identified, Health Insurance Portability and Accountability Act-compliant database of administrative claims for members from the largest US health insurer. All patients meeting the inclusion/exclusion criteria below were included in the study.

All variables in this study, including diseases, clinical procedures, laboratory investigations, medications, and outcomes, were assessed using common International Classification of Diseases (ICD-9/10), procedural (Current Procedural Terminology 4 [CPT-4] or Health Care Common Procedure Coding System), laboratory test (Logical Observation Identifiers Names and Codes), or national drug codes recorded in the database. The categories for these codes are described in Supplementary Table 1.

### Patients

Included patients were indexed by the date when CKD stage 3–4 was identified using ICD-9/10 codes for CKD or laboratory values (estimated glomerular filtration rate [eGFR] 15–59 mL/min/1.73 m^2^) confirmed by a second ICD code or eGFR value between 90 and 365 days apart. In addition, included patients were aged ≥ 18 years at index and had ≥ 365 days of continuous insurance coverage prior to the index event (baseline period). Key exclusion criteria are outlined in the Supplementary Appendix.

The main study cohort included patients with non-diabetic, moderate-to-severe CKD who met all inclusion/exclusion criteria. Two subgroups of this cohort were also analyzed: patients with non-diabetic, moderate-to-severe CKD with hypertension, and patients from the first subgroup who also had coronary artery disease (CAD).

Patient baseline characteristics such as age, gender, comorbidities, and use of comedications were assessed. The index eGFR value and CKD stage were also defined. Details of baseline characterization and assignment of index eGFR and index CKD can be found in the Supplementary Appendix.

### Outcomes

Patients were followed from 1 day after the index event until insurance disenrollment, the end of data availability, death, or the end of the analysis period, whichever occurred first. The primary outcomes were hospitalization for heart failure (HHF), a composite kidney outcome of ESKD/kidney failure (acute and unspecified)/need for dialysis and worsening of CKD stage from baseline. Although guidelines define both ESKD and kidney failure as CKD stage 5 [14], the respective ICD codes used in this study were mutually distinct and did not overlap (Supplementary Table 2). Individual CKD stage was assigned based on eGFR values (priority) or the respective ICD code at index and during the follow-up period. Prespecified secondary CV outcomes included HHF (for incident heart failure), stroke, myocardial infarction, and new onset of atrial fibrillation. Prespecified secondary kidney outcomes included the individual components of the kidney composite, AKI, and relative change in eGFR from baseline (including sustained decreases of ≥ 30%, ≥ 40%, and ≥ 57%). Additionally, a time course analysis of eGFR measurements was performed.

In order to evaluate predictive usefulness of eGFR decline as a marker of hard clinical outcomes in CKD, intercurrent event analyses were conducted to investigate associations between post-baseline eGFR decline of ≥ 30%, ≥ 40%, and ≥ 57% and the risk of the subsequent outcomes of HHF and ESKD/kidney failure/need for dialysis.

### Statistical analyses

Descriptive analyses of the study cohorts at baseline were conducted. Aalen–Johansen cumulative incidence curves were plotted, and cumulative incidence rates and their corresponding hazard ratios and confidence intervals were calculated using the Aalen–Johansen estimator. Incidence rates of clinical outcomes were expressed as number of patients with an event per 100 patient-years (PY) of follow-up. Summary statistics for time-course analysis of metric outcomes were generated for each 3-month period and described using frequencies and rates (events per PY). Intercurrent event analyses were performed in patients with available baseline and 2 follow-up eGFR values, using Cox hazard regression analysis. No adjustments were made based on baseline demographics, comorbidities, or comedications. For full details of how statistical analyses were conducted, see the Supplementary Appendix.

## Results

### Patients

A total of 504,924 patients with non-diabetic CKD were included in the final study cohort (Supplementary Fig. 1).

Of these, 428,867 (84.9%) and 113,239 (22.4%) patients were included in the non-diabetic CKD with hypertension and non-diabetic CKD with hypertension and CAD subgroups, respectively. Baseline characteristics for both subgroups are reported in Supplementary Tables 3 and 4.

In the main cohort, eGFR values on the index date were available for 203,436 (40.3%) patients. A further 20,138 (4.0%) patients, who entered the cohort because of the presence of the CKD diagnosis code on the index date, had available eGFR values in the baseline period. For 281,350 (55.7%) patients, no eGFR value was available in the baseline period; these patients were included in the cohort based on ICD diagnosis codes only.

#### Baseline characteristics

In the main cohort, median age was 75.0 years and most patients were female (60.5%), White (62.5%), and had stage 3 CKD at index (94.7%) (Table 1). Median eGFR at baseline was 53.0 mL/min/1.73 m^2^ and a urine albumin-to-creatinine ratio (UACR) was recorded in 6% of individuals, of whom 73%, 21%, and 6% had normal-to-mildly increased (< 30 mg/g), moderately increased (≥ 30– ≤ 300 mg/g), and severely increased (> 300 mg/g) albuminuria, respectively (Supplementary Fig. 2).

**Table 1:** Baseline demographics, clinical data, comorbidities, and medication use for the main cohort

	Moderate-to-severe non-diabetic CKD (main cohort) *N* = 504,924
Age, years, median (IQR)	75.0 (68.0–81.0)
Gender, *n* (%)	
Female	305,297 (60.5)
Missing	73 (< 0.1)
Race, *n* (%)	
White	315,711 (62.5)
Black	47,947 (9.5)
Unknown	42,339 (8.4)
Hispanic	35,163 (7.0)
Asian	9435 (1.9)
Missing	54,329 (10.8)
Index CKD stage, *n* (%)	
CKD 3	478,415 (94.7)
CKD 4	26,509 (5.3)
Baseline eGFR, mL/min/1.73 m^2^, median (IQR)	53.0 (47.1–57.0) (*n* = 313,367)
Baseline UACR, mg/g, median (IQR)	9.7 (3.0–33.0) (*n* = 30,793)
Baseline UACR, mg/g, *n* (%)	
< 30	22,621 (4.5)
30– ≤ 300	6376 (1.3)
> 300	1796 (0.4)
Missing	474,131 (93.9)
Serum potassium, mmol/L, median (IQR)	4.0 (3.8–4.0) (*n* = 317,505)
Most common comorbidities at baseline, *n* (%)	
Charlson–Deyo comorbidity index, median (IQR)	3.0 (2.0–4.0)
Hypertension	428,867 (84.9)
Hyperlipidemia	344,610 (68.2)
Hypothyroidism	129,616 (25.7)
Anemia	127,423 (25.2)
Pulmonary disease	122,012 (24.2)
CAD	121,368 (24.0)
Heart failure	79,935 (15.8)
Depression and other mood affective disorders	78,353 (15.5)
Specific kidney disease diagnoses, *n* (%)	
Tubulo-interstitial kidney disease	64,135 (12.7)
Glomerulonephritis (chronic or unspecified)	24,728 (4.9)
Polycystic kidney disease	8299 (1.6)
Acute glomerulonephritis	539 (0.1)
IgA nephropathy (Berger’s disease)	423 (0.1)
Most frequently used medications at baseline, *n* (%)	
Centrally acting hypertensive	361,000 (71.5)
Statin	239,015 (47.3)
Beta-blocker	222,183 (44.0)
NSAID	179,762 (35.6)
ACE inhibitor	174,059 (34.5)
Antidepressant	169,682 (33.6)
Thiazide diuretic	117,301 (23.2)
Calcium channel blocker	108,535 (21.5)
ARB	103,871 (20.6)

See Supplementary Table 3 for full data on baseline comorbidities and medication use

*ACE* angiotensin-converting enzyme, *ARB* angiotensin receptor blocker, *CAD* coronary artery disease, *CKD* chronic kidney disease, *eGFR* estimated glomerular filtration rate, *IgA* immunoglobulin A, *IQR* interquartile range, *NSAID* nonsteroidal anti-inflammatory drug, *UACR* urine albumin-to-creatinine ratio

Statins and beta-blockers were used by 47.3% and 44.0% of patients, respectively, and ACEis and ARB therapy were used by 34.5% and 20.6% of patients, respectively. In addition, antidepressants and nonsteroidal anti-inflammatory drugs were used by approximately one-third of patients with non-diabetic CKD (Supplementary Table 3). The diagnosing provider’s specialty was reported on 77.1% of claims for the index event, the most common being internal medicine (18.5%), family medicine (18.0%), clinical medical laboratory (8%), and nephrology (7%) (Supplementary Table 5).

### Outcomes

Out of 504,924 patients with non-diabetic, moderate-to-severe CKD, data of 504,687 patients were available for clinical outcomes analysis over a median follow-up period of 744 (328–1432) days: in total, 182 patients did not start follow-up, and a further 55 patients were excluded because of database inconsistency (date of death was prior to the index date).

#### Primary outcomes and related components

**Fig. 1:**
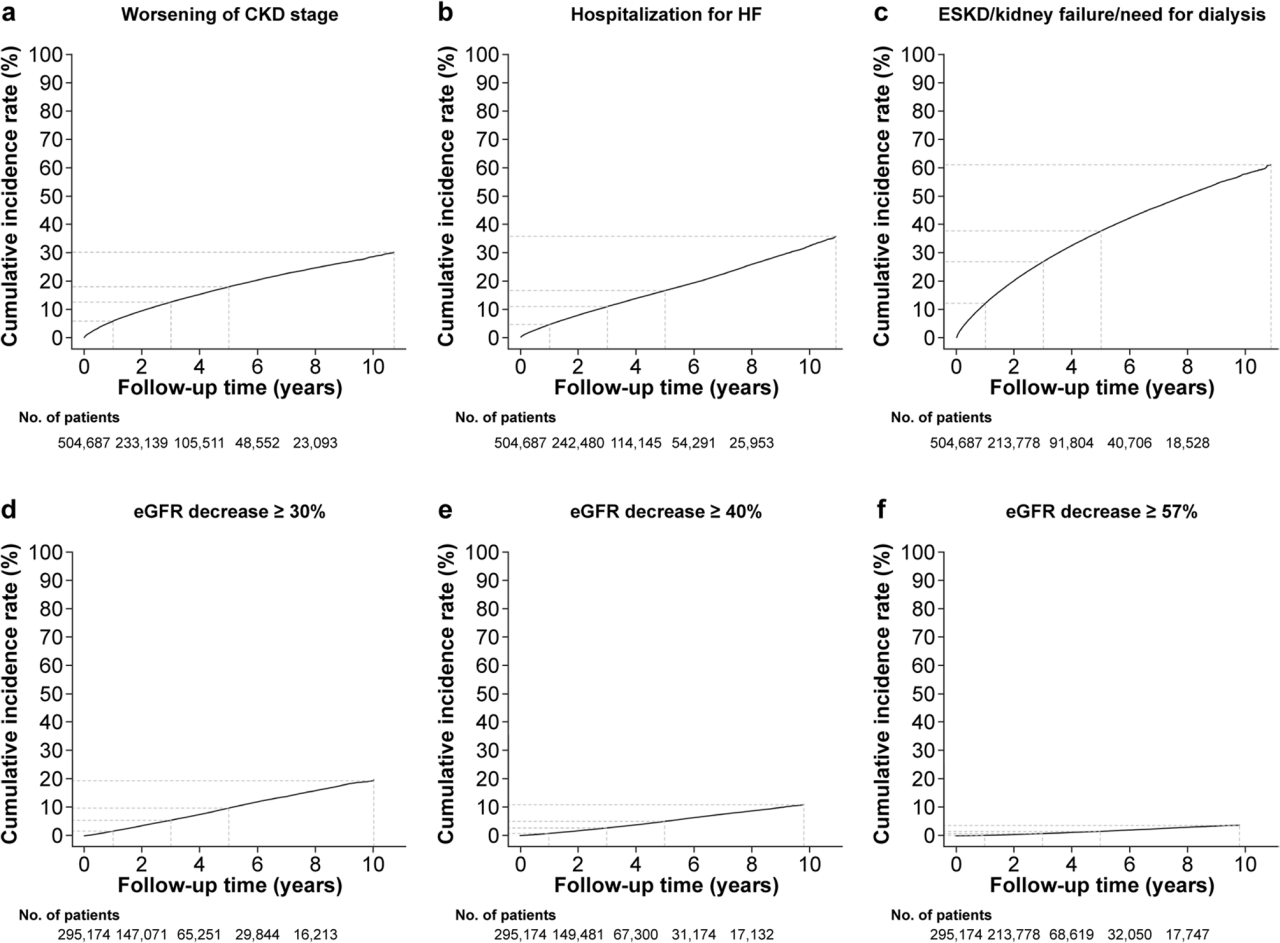
Aalen–Johansen cumulative incidence rate curves for primary outcomes, worsening of CKD stage and eGFR decreases in the main cohort. Worsening of CKD stage from baseline (**a**), HF hospitalization (**b**), ESKD/kidney failure/need for dialysis (**c**), ≥ 30% eGFR decline (**d**); ≥ 40% eGFR decline (**e**), and ≥ 57% eGFR decline (**f**). Dashed lines show the survival rate at 1 year, 3 years, 5 years, and maximum follow-up. *CKD* chronic kidney disease, *eGFR* estimated glomerular filtration rate, *ESKD* end-stage kidney disease, *HF* heart failure

In the main cohort, the incidence rates of the primary outcomes were 4.0 events/100 PY for HHF, 10.3 events/100 PY for the composite kidney outcome and 4.4 events/100 PY for worsening of CKD stage from baseline (Table 2; Fig. 1).

**Table 2:** Clinical outcome incidence rates per 100 PY in the main cohort

	*n*/total (%)	Incidence rate *n*/100 PY
Primary outcomes		
Hospitalization for HF	53,282/504,687 (10.6)	4.0
ESKD/kidney failure/need for dialysis	122,134/504,687 (24.2)	10.3
Worsening of CKD stage	56,330/504,687 (11.2)	4.4
Secondary outcomes		
Cardiovascular		
Hospitalization for incident HF	24,777/401,654 (6.2)	2.2
Stroke	49,332/504,687 (9.8)	3.7
Myocardial infarction	62,123/504,687 (12.3)	4.9
Atrial fibrillation (new onset)	46,780/418,478 (11.2)	4.2
Kidney		
ESKD/need for dialysis	24,259/504,687 (4.8)	1.8
Kidney failure (acute and unspecified)	114,796/504,687 (22.7)	9.5
Need for dialysis	6873/504,687 (1.4)	0.5
Acute kidney injury	106,071/504,687 (21.0)	8.6
Kidney transplant	2833/504,687 (0.6)	0.2
eGFR decrease ≥ 30%	15,823/295,174 (5.4)	2.0
eGFR decrease ≥ 40%	7907/295,174 (2.7)	1.0
eGFR decrease ≥ 57%	2502/295,174 (0.8)	0.3

Incidence rate calculated with the Aalen–Johansen estimator

*CKD* chronic kidney disease, *eGFR* estimated glomerular filtration rate, *ESKD* end-stage kidney disease, *HF* heart failure, *PY* patient-years

Incidence rate of incident HHF was 2.2 events/100 PY. Incidence rates of the components of the kidney composite outcome were 1.8, 9.5, and 0.5 events/100 PY for ESKD/need for dialysis, kidney failure (acute and unspecified), and need for dialysis, respectively (Table 2). Kidney failure events were driven mainly by AKI, with an incidence rate of 8.6 events/100 PY.

An overview of selected outcomes in the hypertension and CAD cohort is presented in Supplementary Table 6. Incidence rates for the primary endpoints were higher in the hypertension and CAD cohort than in the main cohort (Supplementary Table 6 and Supplementary Fig. 3). As the subgroup of patients with non-diabetic CKD with hypertension overlapped substantially with the main study cohort (85% of patients are in both cohorts), the outcomes are not reported for this subgroup.

#### Additional outcomes

Among patients with a baseline eGFR value and a non-zero follow-up time (*n* = 295,174), incidence rates for relative decreases in eGFR of ≥ 30%, ≥ 40%, and ≥ 57% from baseline were 2.0, 1.0, and 0.3 events/100 PY, respectively (Table 2; Fig. 1).

Corresponding incidence rates in the hypertension and CAD cohort are presented in Supplementary Fig. 4.

Incidence rates for a diagnosis of stroke, myocardial infarction, or new-onset atrial fibrillation were 3.7, 4.9, and 4.2 events/100 PY, respectively (Table 2).

#### eGFR time course analysis

For the overall patient cohort, in those with an available baseline eGFR value and a non-zero follow-up time, time-course analysis of eGFR showed no substantial change in eGFR over a 2-year period (Supplementary Table 7). However, in patients with available UACR (*n* = 25,824; < 30 mg/g in 19,030 patients, 30–300 mg/g in 5283 patients, and > 300 mg/g in 1511 patients with a mean eGFR at baseline of 50.69 mL/min/1.73 m^2^, 47.17 mL/min/1.73 m^2^, and 42.79 mL/min/1.73 m^2^, respectively), time–course analysis of eGFR showed that reductions in eGFR mostly occurred in patients with moderately and severely increased albuminuria at baseline (30–300 mg/g and > 300 mg/g, respectively; Supplementary Fig. 5).

#### Intercurrent event analyses

Among patients in the main cohort with a baseline eGFR value and a non-zero follow-up time (*n* = 295,174), eGFR decline was associated with an increased risk of the primary outcomes. The hazard ratio for HHF was 3.61, 3.96, and 4.38 and for ESKD/kidney failure/need for dialysis it was 6.37, 9.17, and 20.21 in patients with eGFR declines of ≥ 30%, ≥ 40%, and ≥ 57%, respectively, compared with those with no such intercurrent event (Table 3; Supplementary Fig. 6).

**Table 3:** Relationship between post-baseline eGFR decline and HHF and ESKD/kidney failure/need for dialysis in the main cohort

eGFR decrease	Outcome	*N* = 295,174	Hazard ratio (95% CI)
		Patients with intercurrent event, *n* (%)	
≥ 30%	HHF	13,798 (4.67)	3.61 (3.45–3.78)
	Composite kidney	9786 (3.32)	6.37 (6.15–6.60)
≥ 40%	HHF	6670 (2.26)	3.96 (3.73–4.21)
	Composite kidney	4093 (1.39)	9.17 (8.67–9.70)
≥ 57%	HHF	2076 (0.70)	4.38 (3.94–4.86)
	Composite Kidney	841 (0.28)	20.21 (17.30–23.60)

Decrease in eGFR was assessed across the follow-up period between the index date and the outcome. Hazard ratio shows the risk of each outcome in patients with a given decrease in eGFR compared with patients who did not experience the given decrease in eGFR

*eGFR* estimated glomerular filtration rate, *ESKD* end-stage kidney disease, *HHF* hospitalization for heart failure

## Discussion

This study generated real-world evidence (RWE) on baseline demographics, clinical characteristics, and clinical outcomes in individuals with moderate-to-severe non-diabetic CKD treated in routine clinical practice in the US. RWE provides observational insights that are often used, in combination with clinical trial evidence, to support patient care and decision-making in routine clinical practice [15, 16].

Overall, 60% of patients in the study were women, which is consistent with the US Renal Data System and the CURE-CKD patient population (both include patients with diabetes) [17, 18], but notably higher than the non-diabetic cohort of the Dapagliflozin and Prevention of Adverse Outcomes in Chronic Kidney Disease (DAPA-CKD) trial (33% female), which suggests under-representation of female patients in clinical trials[19]. Despite the higher number of female patients in our study, RWE suggests that more male patients are treated with dialysis; in the US Renal Data System, the adjusted ESKD incidence was 63% higher in men than women in 2018 [20]. Investigation into the causes of potential sex-related differences in CKD progression may be warranted.

In this study, despite a high prevalence of classic risk factors for CKD, screening for CKD was under-reported. At baseline, 38% and ~ 94% of patients had no eGFR or UACR recorded, respectively, despite CKD guidelines recommending at least annual eGFR and UACR assessment in high-risk patients [14]. Under-reporting and underdiagnosis is a challenge in CKD management [18]. Further research and education are needed to improve awareness and early diagnosis of CKD.

Our results indicate that patients are generally undertreated with kidney and CV medications. ACEi/ARB use was low (34.5% and 20.6%, respectively) given that patients had CKD and the Kidney Disease Improving Global Outcomes guidelines recommend use of these therapies in patients with UACR > 300 mg/g; however, of the limited number of patients with available UACR values (6.1%), most had a UACR < 300 mg/g. Analysis from the CURE-CKD registry reported that a renin–angiotensin system inhibitor was prescribed to just 20.6% of patients overall, and to 20.5% of patients with CKD and hypertension [18]. Statin and antiplatelet use was low in the CKD plus hypertension and CAD cohort (47.3% and 7.2%, respectively) despite findings from the SHARP trial and Kidney Disease Improving Global Outcomes lipid management guidelines, which recommend use of these therapies in this patient population [14, 21, 22]. The recorded low use of CV and kidney-protective agents reflects the potential to improve standard of care in clinical practice. Lastly, in accordance with the CURE-CKD registry analysis, the current analysis shows that there is a high use of nonsteroidal anti-inflammatory drugs among patients with CKD (35.6%) despite known kidney risks associated with this medication class [14, 18].

The intercurrent event analysis demonstrated that the risk of kidney and CV events increases with the increasing magnitude of the post-baseline eGFR decline. There is still an urgent need to identify surrogate markers in CKD that may accurately predict hard clinical outcomes, such as ESKD, which may take years to be reached [23]. The US Food and Drug Administration has historically accepted the doubling of serum creatinine as a surrogate endpoint for the development of kidney failure (relating to ~ 57% decline in eGFR), and has also been open to the use of lower levels of eGFR decline to detect earlier stages of kidney disease progression (with the Finerenone in Reducing Kidney Failure and Disease Progression in Diabetic Kidney Disease [FIDELIO-DKD] and EMPA-KIDNEY [The Study of Heart and Kidney Protection With Empagliflozin] randomized controlled trials including a ≥ 40% eGFR decline as a primary endpoint component) [12, 23, 24]. Our findings support the hypothesis that a 30% or 40% decline in eGFR can identify patients at higher risk of cardiorenal events in a real-world setting. In addition, a meta-analysis of treatment effects of randomized controlled trials showed that the mean rate of change in eGFR (eGFR slope) is also a strong surrogate marker of kidney failure [25].

This analysis shows that real-world patients with non-diabetic CKD are at high risk of serious clinical outcomes, including HHF, ESKD/kidney failure/need for dialysis, AKI, and CKD progression. There is currently limited RWE specifically relating to non-diabetic cohorts, with most CKD RWE studies including patients with mixed etiologies. This highlights the value of our analysis and the need for further studies focusing on this patient population.

### Study strengths and limitations

This study investigated a large patient cohort comprising more than 500,000 patients with incident and prevalent non-diabetic, moderate-to-severe CKD. The majority of patients were included in the cohort using ICD codes since laboratory data was only available in 30% of patients. Although this approach has limitations, it has been shown that the code-based definition of CKD stage 3–4 using claims databases has a positive predictive value of > 80% [26]. Therefore, it can be assumed that the findings of the study are not dependent on the method used for inclusion of patients in the cohort.

Considering the nature of CKD and the high rate of undiagnosed patients in the early stages of the disease, it was not possible to identify genuine new cases. This is a limitation that needs to be accounted for when interpreting the results of the study. However, the inclusion of patients with moderate-to-severe CKD enabled assessment of clinical outcomes and monitoring of CKD progression that are known to happen in late stages of this disease.

The study is based on a database of claims from a single US health insurer, so the reimbursement data may not fully represent the whole of the US, and the levels of screening for CKD may not reflect those in clinical practice. Treatment usage may be underestimated in this cohort because of the increasing use of high deductible health plans and low-cost generic programs in the United States; the database would not have captured any drugs dispensed to patients outside of their insurance plan. In contrast, treatment use may also be overestimated because it is not known whether the medication dispensed was actually taken by the patient. In addition, the short follow-up time (average enrollment time in the database was ~ 3 years) may not have been long enough to follow progression from moderate-to-severe CKD to ESKD in all patients.

## Conclusions

The FLIEDER study, comprising a large cohort of patients (> 500,000) from a US database of administrative claims, showed that real-world patients with incident and prevalent non-diabetic, moderate-to-severe CKD are at high risk of serious CV and adverse kidney outcomes. Furthermore, screening for CKD is under-reported and patients are generally undertreated with kidney and CV medications. Our results highlight the high unmet medical need and the urgency for new treatments and targeted interventions for patients with non-diabetic CKD.

## Supplementary Information

Below is the link to the electronic supplementary material.

Supplementary file1 (DOCX 897 kb)

## Data Availability

Data supporting the current study were used under Bayer license agreement with Optum Insights, and as such, are not publicly available. Data are, however, available from the authors upon reasonable request and with permission from Optum Insights. Complete FLIEDER study documentation is a property of Bayer AG and can be provided upon request.

## Declarations

### Study design

This analysis was a retrospective, observational, real-world study using a secondary data source.
