# Supplementary material for: Clinical characteristics and disease outcomes in non-diabetic chronic kidney disease: retrospective analysis of a US healthcare claims database
Source: J Nephrol. 2022 May 14;36(1):45–54. doi: 10.1007/s40620-022-01340-x (PMC9895008; doi:10.1007/s40620-022-01340-x)
Supplement: Supplementary file 1 — Supplementary file1 (DOCX 897 kb) [file 40620_2022_1340_MOESM1_ESM.docx]

**Clinical characteristics and disease outcomes in non-diabetic chronic kidney disease: Retrospective analysis of a US healthcare claims database**

Christoph Wanner, MD^1^, Johannes Schuchhardt, PhD^2^, Chris Bauer, PhD^2^, Stefanie Lindemann, MD^3^, Meike Brinker, MD^4^, Sheldon X Kong, PhD^5^, Frank Kleinjung, PhD^3^, Andrea Horvat-Broecker, MD^4^, Tatsiana Vaitsiakhovich, PhD^3^

^1^Medizinische Klinik und Poliklinik 1, Schwerpunkt Nephrologie, Universitätsklinik Würzburg, Germany

^2^MicroDiscovery GmbH, Berlin, Germany

^3^Bayer AG, Berlin, Germany

^4^Bayer AG, Wuppertal, Germany

^5^Bayer Pharmaceuticals, Whippany, NJ, USA

Supplementary appendix

Additional methods

*CKD stages*

Estimated glomerular filtration rate (eGFR) categories in chronic kidney disease (CKD) were assigned according to the categories outlined in Supplementary Table 8. In the absence of evidence of kidney damage, neither eGFR category G1 nor G2 fulfill the criteria for CKD. Albuminuria categories in CKD were assigned according to the categories outlined in Supplementary Table 8. Categorization of CKD based on GFR and albuminuria was based on Kidney Disease Outcomes Quality Initiative 2002 classifications (Supplementary Table 8).

*Exclusion criteria*

Patients were excluded from the study if they:

- Were diagnosed with diabetes mellitus (based on International Classification of Diseases [ICD] codes) or had severe kidney disease, including CKD stage 5 or end-stage kidney disease prior to the index date
- Had a kidney transplant, kidney failure (acute or unspecified), or dialysis in the 365-day baseline period

*Baseline characterization and assignment of index eGFR value and index CKD*

Patient baseline characteristics such as age, gender, comorbidities, and use of comedications were assessed at the index date or from the last recorded value within the baseline period for the study cohorts. The Charlson-Deyo Comorbidity Index of classifying comorbid conditions that alter the risk of mortality was calculated. This index is based on 17 clinical conditions and ranges from 0 (no comorbidities) to 29 (maximal disease burden),^1,2^ with all patients in this study having a minimum score of 2 due to the presence of CKD stage 3 or 4.

For patients included in the study, the index eGFR value and index CKD stage were defined in one of four ways:

1. In cases where patients were selected based on 2 eGFR measurements, the second eGFR measurement determined the index eGFR value and the corresponding CKD stage
2. In cases where patients were selected based on an ICD code followed by an eGFR measurement, the eGFR measurement determined the index eGFR value and the corresponding CKD stage
3. In cases where patients were selected based on an eGFR measurement followed by an ICD code, the index eGFR value was defined as the value of the eGFR measurement taken during the baseline period between the date of the first eGFR measurement (used as the selection criterion) and the index date, if available; if not, the first eGFR measurement was used. The index CKD stage was defined as per the index ICD code
4. In cases where patients were selected based on two ICD codes for CKD stage 3 or 4, the index eGFR value was defined as the value of the eGFR measurement taken during the baseline period between the date of the first ICD code and the index date, if available; otherwise, the index eGFR was not defined. The index CKD stage was defined as per the index ICD code.

*Outcomes*

Worsening of CKD stage was analyzed based on the patient index CKD stage and the CKD stage in the follow-up period, which was assigned based either on eGFR (priority) or ICD code for CKD. eGFR measurement or ICD diagnosis code indicating more severe CKD stage as compared to the index CKD stage in the follow-up period indicated the outcome “worsening of CKD stage.”

*Statistical analysis*

Descriptive analyses of the study cohorts at baseline were conducted using R version 3.6.2. The results are described by presenting frequency distributions and basic summary statistics. Results for categorical variables are reported by absolute and relative frequencies and results for continuous variables are given by sample statistics.

For the intercurrent event analysis, an intercurrent event was defined as an event that occurred after a given index event and either precluded an observation of a variable of interest (e.g., clinical outcome) or affected its interpretation. In the context of this study the association between eGFR decline of ≥ 30%, ≥ 40%, and ≥ 57% post-index and the risk of HHF and the composite kidney outcome was analyzed. The aforementioned variables were selected *a priori* for the purposes of this analysis.

The analysis of a potential effect of the intercurrent event on the outcomes was performed by the Cox hazard regression method in R package “rms” version 5.1-4 for the following derived time-to-event data sets, (see b):

- “First period data set”: patient data were used from the start of the follow-up period until the end of the follow-up period, if no intercurrent event occurred; otherwise, until the date of the first intercurrent event

Patients were right censored at the first intercurrent event

- “Second period data set”: patient data were used from the date of the first intercurrent event (patients without intercurrent events were neglected) until the end of the follow-up period, if exactly one intercurrent event was present; otherwise, until the date of the second intercurrent event

Patients were right censored at the second intercurrent event

This scheme could be generally applied for as many periods as reasonable.

References

1. Deyo RA, Cherkin DC, Ciol MA (1992) Adapting a clinical comorbidity index for use with ICD-9-CM administrative databases. J Clin Epidemiol 45:613–9.

2. Quan H, Sundararajan V, Halfon P, et al (2005) Coding algorithms for defining comorbidities in ICD-9-CM and ICD-10 administrative data. Med Care 43:1130–9.

Supplementary Tables and Figures

**Supplementary Table 1** Variables in the study that were assessed using clinical codes entered into the database

| **Comorbidities/outcomes*** |
| --- |
| Acute glomerulonephritis |
| Acute coronary syndrome |
| Acute kidney injury/failure |
| Alcohol abuse |
| Allergic reaction |
| Anemia |
| Angina |
| Aortic plaque |
| Arteritis |
| Ataxia |
| Atherosclerosis |
| Atrial fibrillation |
| CDCI - any malignancy, including lymphoma and leukemia, except malignant neoplasms of skin |
| CDCI - cerebrovascular disease |
| CDCI - chronic pulmonary disease |
| CDCI - congestive heart failure |
| CDCI - dementia |
| CDCI - diabetes with chronic complication |
| CDCI - diabetes without chronic complication |
| CDCI - hemiplegia or paraplegia |
| CDCI - HIV/AIDS |
| CDCI - metastatic solid tumor |
| CDCI - mild liver disease |
| CDCI - moderate or severe liver disease |
| CDCI - myocardial infarction |
| CDCI - peptic ulcer disease |
| CDCI - peripheral vascular disease |
| CDCI - renal disease |
| CDCI - rheumatic disease |
| CKD stage 1 |
| CKD stage 2 |
| CKD stage 3 |
| CKD stage 4 |
| CKD stage 5/ESRD/without dialysis |
| Connective tissue disorders |
| Coronary artery disease |
| Dementia |
| Depression and other mood affective disorders |
| Diabetic retinopathy |
| Dialysis |
| Diabetes mellitus all types |
| Drug abuse |
| Edema |
| Gastric or peptic ulcer |
| Genital issues |
| Glomerulonephritis chronic or unspecified |
| Gynecomastia |
| Heart failure |
| History of major bleeding |
| HIV/AIDS |
| Hodgkin's disease |
| Hyperaldosteronism |
| Hyperkalemia |
| Hyperlipidemia |
| Hypertension |
| Hyperthyroidism |
| Hypoglycemia |
| Hypokalemia |
| Hypotension |
| Hypothyroidism |
| IgA nephropathy (Berger’s disease) |
| Inflammatory bowel disease |
| Intracranial hemorrhage |
| Ischemic stroke |
| Kidney transplant |
| Liver disease |
| Lymphoid leukemia |
| Major bleeding |
| Malignant neoplasm |
| Mechanical heart valve |
| Microvascular complications |
| Mitral stenosis |
| Monocytic leukemia |
| Multiple myeloma |
| Myasthenia gravis |
| Myeloid leukemia |
| Myocardial infarction |
| Nephritic syndrome |
| Nephritis |
| Nephropathy |
| Nephrotic syndrome |
| Neuroendocrine tumors |
| Neuropathy |
| Obesity |
| Obstructive and reflux uropathy or urolithiasis |
| Peripheral arterial disease |
| Polycystic kidney disease |
| Proteinuria |
| Psychosis |
| Pulmonary disease |
| Pyelonephritis |
| Renal agenesis, dysgenesis, and hypoplasia |
| Renal artery occlusion or renal artery stenosis |
| Renal tubulointerstitial diseases |
| Rheumatic disease |
| Sleep apnea |
| Systemic lupus erythematosus and antineutrophil cytoplasmic antibody (ANCA) associated vasculitis (SLE/AAV) |
| Thrombosis |
| Tobacco abuse |
| Transient ischemic attack |
| Tubular necrosis |
| Tumor |
| Unspecified and acute kidney failure |
| Unspecified kidney failure |
| Vascular disease |
| **Procedures*** |
| Amputation (diabetic) |
| Angioplasty |
| Aortic repair |
| Coronary artery bypass grafts (CABG) |
| Laparotomy with small or large bowel restriction |
| Transcatheter aortic valve replacement |
| Revascularization for CAD |
| Surgical aortic valve replacement |
| Stent Implantation |
| **Comedication^†^** |
| Alpha blocker |
| ACE inhibitor |
| ARB therapy |
| ARNI |
| Antiarrhythmics |
| Antidepressants |
| Antiplatelet therapy |
| Beta blockers |
| Biguanides |
| Calcium channel blockers |
| Central acting hypertensive |
| Direct renin Inhibitors |
| DPP4 Inhibitors |
| Epithelial sodium channel blockers |
| GLP-1RA |
| Insulin |
| NSAIDs |
| Oral anticoagulants |
| Potassium binding agents |
| Potassium supplement |
| SGLT-2 inhibitors |
| Statin |
| Sulfonylureas |
| Vasodilators |

*CPT/HCPCS, ICD-10-CM, ICD-10-PCS, ICD-9-CM, and ICD-9-PCS codes

^†^NDC codes

AAV, ANCA-associated vasculitis; ACE, angiotensin-converting enzyme; AIDS, acquired immunodeficiency syndrome; ANCA, antineutrophil cytoplasmic antibody; ARB, angiotensin receptor blocker; ARNI, angiotensin receptor neprilysin inhibitor; CABG, coronary artery bypass graft; CAD, coronary artery disease; CDCI, Charlson-Deyo Comorbidity Index; CKD, chronic kidney disease; CM, clinical modification developed in US; CPT, current procedural terminology; DPP4, dipeptidyl-peptidase 4; ESRD, end-stage renal disease; GLP-1RA, glucagon-like peptide-1 receptor agonist; HCPCS, healthcare common procedure coding system; HIV, human immunodeficiency virus; ICD, International Classification of Diseases; IgA, immunoglobulin A; NDC, national drug code; NSAID, nonsteroidal anti-inflammatory drug; PCS, procedure coding system; SGLT-2, sodium-glucose co-transporter-2; SLE, systemic lupus erythematosus

**Supplementary Table 2** Definition of composite kidney outcome components by clinical codes

| **Code** | **Description** |
| --- | --- |
| **ESKD** |  |
| **ICD-9-CM** |  |
| 403.01 | Hypertensive chronic kidney disease malignant with chronic kidney disease stage V or end stage renal disease |
| 403.91 | Hypertensive chronic kidney disease unspecified with chronic kidney disease stage V or end stage renal disease |
| 585.5 | Chronic kidney disease stage V |
| 585.6 | End stage renal disease |
| **ICD-10-CM** |  |
| N18.6 | End stage renal disease |
| I12.0 | Hypertensive chronic kidney disease with stage 5 chronic kidney disease or end stage renal disease |
| I13.2 | Hypertensive heart and chronic kidney disease with heart failure and with stage 5 chronic kidney disease, or end stage renal disease |
| I13.11 | Hypertensive heart and chronic kidney disease without heart failure, with stage 5 chronic kidney disease, or end stage renal disease |
| N18.5 | Chronic kidney disease, stage 5 |
| **Kidney failure** |  |
| **ICD-9-CM** |  |
| 586 | Renal failure unspecified |
| 584.5 | Acute kidney failure with lesion of tubular necrosis |
| 584.6 | Acute kidney failure with lesion of renal cortical necrosis |
| 584.7 | Acute kidney failure with lesion of renal medullary (papillary) necrosis |
| 584.8 | Acute kidney failure with other specified pathological lesion in kidney |
| 584.9 | Acute kidney failure unspecified |
| **ICD-10-CM** |  |
| N19 | Unspecified kidney failure |
| N17 | Acute kidney failure |
| N17.0 | Acute kidney failure with tubular necrosis |
| N17.1 | Acute kidney failure with acute cortical necrosis |
| N17.2 | Acute kidney failure with medullary necrosis |
| N17.8 | Other acute kidney failure |
| N17.9 | Acute kidney failure, unspecified |
| **Dialysis** |  |
| **ICD-9-CM** |  |
| V45.12 | Noncompliance with renal dialysis |
| V56.3 | Encounter for adequacy testing for dialysis |
| V56.8 | Aftercare involving other dialysis |
| V56 | Encounter for dialysis and dialysis catheter care |
| V56.2 | Fitting and adjustment of peritoneal dialysis catheter |
| V56.0 | Aftercare involving extracorporeal dialysis |
| V56.31 | Encounter for adequacy testing for hemodialysis |
| V56.32 | Encounter for adequacy testing for peritoneal dialysis |
| V45.11 | Renal dialysis status |
| V56.1 | Fitting and adjustment of extracorporeal dialysis catheter |
| V45.1 | Renal dialysis status |
| **ICD-10-CM** |  |
| R88.0 | Cloudy (hemodialysis) (peritoneal) dialysis effluent |
| T81.502 | Unspecified complication of foreign body accidentally left in body following kidney dialysis |
| T81.502D | Unspecified complication of foreign body accidentally left in body following kidney dialysis, subsequent encounter |
| T82.41XS | Breakdown (mechanical) of vascular dialysis catheter, sequela |
| T82.43XS | Leakage of vascular dialysis catheter, sequela |
| T85.621S | Displacement of intraperitoneal dialysis catheter, sequela |
| T85.71 | Infection and inflammatory reaction due to peritoneal dialysis catheter |
| Z49.3 | Encounter for adequacy testing for dialysis |
| Z49.32 | Encounter for adequacy testing for peritoneal dialysis |
| I95.3 | Hypotension of hemodialysis |
| T82.42 | Displacement of vascular dialysis catheter |
| T82.42XA | Displacement of vascular dialysis catheter, initial encounter |
| T82.42XS | Displacement of vascular dialysis catheter, sequela |
| T82.43XA | Leakage of vascular dialysis catheter, initial encounter |
| T82.49 | Other complication of vascular dialysis catheter |
| T85.611A | Breakdown (mechanical) of intraperitoneal dialysis catheter, initial encounter |
| T85.621D | Displacement of intraperitoneal dialysis catheter, subsequent encounter |
| T85.631A | Leakage of intraperitoneal dialysis catheter, initial encounter |
| T85.71XD | Infection and inflammatory reaction due to peritoneal dialysis catheter, subsequent encounter |
| Z49 | Encounter for care involving renal dialysis |
| Z91.15 | Patient's noncompliance with renal dialysis |
| T81.502S | Unspecified complication of foreign body accidentally left in body following kidney dialysis, sequela |
| T82.41XD | Breakdown (mechanical) of vascular dialysis catheter, subsequent encounter |
| T82.42XD | Displacement of vascular dialysis catheter, subsequent encounter |
| T82.49XA | Other complication of vascular dialysis catheter, initial encounter |
| T85.621 | Displacement of intraperitoneal dialysis catheter |
| T85.631 | Leakage of intraperitoneal dialysis catheter |
| T85.71XA | Infection and inflammatory reaction due to peritoneal dialysis catheter, initial encounter |
| Y84.1 | Kidney dialysis as the cause of abnormal reaction of the patient, or of later complication, without mention of misadventure at the time of the procedure |
| Z49.0 | Preparatory care for renal dialysis |
| Z49.01 | Encounter for fitting and adjustment of extracorporeal dialysis catheter |
| T81.502A | Unspecified complication of foreign body accidentally left in body following kidney dialysis, initial encounter |
| T82.4 | Mechanical complication of vascular dialysis catheter |
| T82.43 | Leakage of vascular dialysis catheter |
| T82.49XD | Other complication of vascular dialysis catheter, subsequent encounter |
| T85.621A | Displacement of intraperitoneal dialysis catheter, initial encounter |
| T85.691 | Other mechanical complication of intraperitoneal dialysis catheter |
| T85.691A | Other mechanical complication of intraperitoneal dialysis catheter, initial encounter |
| T85.691D | Other mechanical complication of intraperitoneal dialysis catheter, subsequent encounter |
| T85.691S | Other mechanical complication of intraperitoneal dialysis catheter, sequela |
| Y62.2 | Failure of sterile precautions during kidney dialysis and other perfusion |
| Z49.31 | Encounter for adequacy testing for hemodialysis |
| T82.41 | Breakdown (mechanical) of vascular dialysis catheter |
| T82.41XA | Breakdown (mechanical) of vascular dialysis catheter, initial encounter |
| T82.43XD | Leakage of vascular dialysis catheter, subsequent encounter |
| T82.49XS | Other complication of vascular dialysis catheter, sequela |
| T85.611 | Breakdown (mechanical) of intraperitoneal dialysis catheter |
| T85.611D | Breakdown (mechanical) of intraperitoneal dialysis catheter, subsequent encounter |
| T85.611S | Breakdown (mechanical) of intraperitoneal dialysis catheter, sequela |
| T85.631D | Leakage of intraperitoneal dialysis catheter, subsequent encounter |
| T85.631S | Leakage of intraperitoneal dialysis catheter, sequela |
| T85.71XS | Infection and inflammatory reaction due to peritoneal dialysis catheter, sequela |
| Z49.02 | Encounter for fitting and adjustment of peritoneal dialysis catheter |
| **ICD-10-PCS** |  |
| B50W0ZZ | Plain radiography of dialysis shunt/fistula using high osmolar contrast |
| B51WZZZ | Fluoroscopy of dialysis shunt/fistula |
| B51W0ZA | Fluoroscopy of dialysis shunt/fistula using high osmolar contrast, guidance |
| B51WYZZ | Fluoroscopy of dialysis shunt/fistula using other contrast |
| B51WZZA | Fluoroscopy of dialysis shunt/fistula, guidance |
| B50W1ZZ | Plain radiography of dialysis shunt/fistula using low osmolar contrast |
| B50WYZZ | Plain radiography of dialysis shunt/fistula using other contrast |
| B51W0ZZ | Fluoroscopy of dialysis shunt/fistula using high osmolar contrast |
| B51W1ZA | Fluoroscopy of dialysis shunt/fistula using low osmolar contrast, guidance |
| B51W1ZZ | Fluoroscopy of dialysis shunt/fistula using low osmolar contrast |
| **CPT-4** |  |
| 0507F | Peritoneal dialysis plan of care documented |
| 4053F | Hemodialysis via functioning arterio-venous (AV) graft (ESRD) / Hemodialysis via functioning arteriovenous (AV) graft (ESRD) |
| 4055F | Patient receiving peritoneal dialysis |
| 90937 | Hemodialysis procedure requiring repeated evaluation(s) with or without substantial revision of dialysis prescription |
| 90989 | Dialysis training, patient, including helper where applicable, any mode, completed course |
| 4054F | Hemodialysis via catheter (ESRD) |
| 75791 | Angiography, arteriovenous shunt (eg, dialysis patient fistula/graft), complete evaluation of dialysis access, including fluoroscopy, image documentation and report (includes injections of contrast and all necessary imaging from the arterial anastomosis and adjacent artery through entire venous outflow including the inferior or superior vena cava), radiological supervision and interpretation |
| 90947 | Dialysis procedure other than hemodialysis (eg, peritoneal dialysis, hemofiltration, or other continuous renal replacement therapies) requiring repeated evaluation by a physician or other qualified health care professional, with or without substantial revision of dialysis prescription / Dialysis procedure other than hemodialysis (eg, peritoneal dialysis, hemofiltration, or other continuous renal replacement therapies) requiring repeated evaluations by a physician or other qualified health care professional, with or without substantial revision of dialysis prescription / Dialysis procedure other than hemodialysis (eg, peritoneal dialysis, hemofiltration, or other continuous renal replacement therapies) requiring repeated physician evaluations, with or without substantial revision of dialysis prescription / Dialysis procedure other than hemodialysis (eg, peritoneal, hemofiltration) requiring repeated evaluations, with or without substantial revision of dialysis prescription |
| 90993 | Dialysis training, patient, including helper where applicable, any mode, course not completed, per training session |
| 0505F | Hemodialysis plan of care documented (ESRD) / Hemodialysis plan of care documented (ESRD, P-ESRD) |
| 90939 | Hemodialysis access flow study to determine blood flow in grafts and arteriovenous fistulae by an indicator dilution method, hook-up; transcutaneous measurement and disconnection |
| 90999 | Unlisted dialysis procedure, inpatient or outpatient |
| 90940 | Hemodialysis access flow study to determine blood flow in grafts and arteriovenous fistulae by an indicator dilution method, hook-up, measurement and disconnection / Hemodialysis access flow study to determine blood flow in grafts and arteriovenous fistulae by an indicator dilution method, hook-up; measurement and disconnection / Hemodialysis access flow study to determine blood flow in grafts and arteriovenous fistulae by an indicator method |
| 90945 | Dialysis procedure other than hemodialysis (eg, peritoneal dialysis, hemofiltration, or other continuous renal replacement therapies), with single evaluation by a physician or other qualified health care professional / Dialysis procedure other than hemodialysis (eg, peritoneal dialysis, hemofiltration, or other continuous renal replacement therapies), with single physician evaluation / Dialysis procedure other than hemodialysis (eg, peritoneal, hemofiltration), with single physician evaluation |
| 99559 | Home infusion of peritoneal dialysis, per diem / Home infusion of peritoneal dialysis, per visit |
| 4052F | Hemodialysis via functioning arterio-venous (AV) fistula (ESRD) / Hemodialysis via functioning arteriovenous (AV) fistula (ESRD) |
| 90935 | Hemodialysis procedure with single evaluation by a physician or other qualified health care professional / Hemodialysis procedure with single physician evaluation |
| 99512 | Home visit for hemodialysis / Home visit for hemodialysis, per diem |
| A4663 | Blood pressure cuff only |
| A4690 | Dialyzer (artificial kidneys), all types, all sizes, for hemodialysis, each |
| A4700 | Standard dialysate solution, each |
| A4726 | Dialysate solution, any concentration of dextrose, fluid volume greater than 5999cc, for peritoneal dialysis |
| A4728 | Dialysate solution, non-dextrose containing, 500 ml |
| A4820 | Hemodialysis kit supplies |
| A4910 | Non-medical supplies for dialysis, (i.e., scale, scissors, stopwatch, etc.) |
| A4913 | Miscellaneous dialysis supplies, not otherwise specified |
| E1638 | Heating pad, for peritoneal dialysis, any size, each |
| G0257 | Unscheduled or emergency dialysis treatment for an ESRD patient in a hospital outpatient department that is not certified as an ESRD facility |
| J0887 | Injection, epoetin beta, 1 microgram, (for ESRD on dialysis) |
| Q4054 | Injection, darbepoetin alfa, 1 mcg (for ESRD on dialysis) |
| Q4081 | Injection, epoetin alfa, 100 units (for ESRD on dialysis) |
| A4655 | Needles and syringes for dialysis |
| A4672 | Drainage extension line, sterile, for dialysis, each |
| A4705 | Bicarbonate dialysate solution, each |
| A4722 | Dialysate solution, any concentration of dextrose, fluid volume greater than 1999cc but less than or equal to 2999cc, for peritoneal dialysis |
| A4760 | Dialysate solution test kit, for peritoneal dialysis, any type, each |
| C1750 | Catheter, hemodialysis, long-term / catheter, hemodialysis/peritoneal, long-term |
| E1592 | Automatic intermittent peritoneal dialysis system |
| E1699 | Dialysis equipment, not otherwise specified |
| A4725 | Dialysate solution, any concentration of dextrose, fluid volume greater than 4999cc but less than or equal to 5999cc, for peritoneal dialysis |
| A4765 | Dialysate concentrate, powder, additive for peritoneal dialysis, per packet |
| A4766 | Dialysate concentrate, solution, additive for peritoneal dialysis, per 10 ml |
| A4919 | No longer used |
| C1037 | Catheter, vaxcel chronic dialysis catheter, medcomp bio flex tesio catheter, medcomp silicone tesio catheter, medcomp hemo-cath long term silicone catheter, bard niagara dual lumen catheter, bard opti-flow dual lumen catheter, medcomp ash split catheter |
| E1590 | Hemodialysis machine |
| E1634 | Peritoneal dialysis clamps, each |
| E1635 | Compact (portable) travel hemodialyzer system |
| E1639 | Scale, each |
| J0886 | Injection, epoetin alfa, 1000 units (for ESRD on dialysis) |
| Q4055 | Injection, epoetin alfa, 1000 units (for ESRD on dialysis) |
| A4721 | Dialysate solution, any concentration of dextrose, fluid volume greater than 999cc but less than or equal to 1999cc, for peritoneal dialysis |
| A4780 | Sterilizing agent for dialysis equipment, per gallon |
| A4800 | Heparin for dialysis and antidote, any strength, porcine or beef, up to 1000 units, 10-30 ml (for parenteral use see b4216) |
| A4929 | Tourniquet for dialysis, each |
| C1752 | Catheter, hemodialysis, short-term / catheter, hemodialysis/peritoneal, short-term |
| E1510 | Kidney, dialysate delivery syst. kidney machine, pump recirculating, air removal syst, flowrate meter, power off, heater and temperature control with alarm, i.v. poles, pressure gauge, concentrate container / kidney, dialysate delivery syst. kidney machine, pump recirculating, air removal syst, flowrate meter, power off, heater and temperature control with alarm, i.v. poles, pressure gauge, concentrate container / kidney, dialysate delivery syst. kidney machine, pump recirculating, air removal syst, flowrate meter, power off, heater and temperature control with alarm, i.v. poles, pressure gauge, concentrate container |
| K0610 | Peritoneal dialysis clamps, each |
| K0612 | Drainage extension line, sterile, for dialysis, each |
| Q0139 | Injection, ferumoxytol, for treatment of iron deficiency anemia, 1 mg (for ESRD on dialysis) |
| S9339 | Home therapy; peritoneal dialysis, administrative services, professional pharmacy services, care coordination and all necessary supplies and equipment (drugs and nursing visits coded separately), per diem / home therapy; peritoneal dialysis, administrative services, professional pharmacy services, care coordination and all necessary supplies and equipment (drugs and nursing visits coded separately), per diem |
| A4720 | Dialysate solution, any concentration of dextrose, fluid volume greater than 249cc, but less than or equal to 999cc, for peritoneal dialysis |
| A4723 | Dialysate solution, any concentration of dextrose, fluid volume greater than 2999cc but less than or equal to 3999cc, for peritoneal dialysis |
| A4724 | Dialysate solution, any concentration of dextrose, fluid volume greater than 3999cc but less than or equal to 4999cc, for peritoneal dialysis |
| A4790 | Cleansing agents for equipment for dialysis only |
| C1881 | Dialysis access system (implantable) |
| E1594 | Cycler dialysis machine for peritoneal dialysis |
| E1637 | Hemostats, each |
| G0324 | End stage renal disease (ESRD) related services for home dialysis (less than full month), per day; for patients under two years of age / end stage renal disease (ESRD) related services less than full month, per day; for patients under two years of age |
| G0325 | End stage renal disease (ESRD) related services for home dialysis (less than full month), per day; for patients between two and eleven years of age / end stage renal disease (ESRD) related services less than full month, per day; for patients between two and eleven years of age |
| G8075 | End stage renal disease patient with documented dialysis dose of URR greater than or equal to 65% (or kt/v greater than or equal to 1. 2) / end stage renal disease patient with documented dialysis dose of URR greater than or equal to 65% (or kt/v greater than or equal to 1.2) |
| G8082 | End stage renal disease patient requiring hemodialysis documented to have received vascular access other than autogenous AV fistula |
| J0882 | Injection, darbepoetin alfa, 1 microgram (for ESRD on dialysis) |
| S9335 | Home therapy, hemodialysis; administrative services, professional pharmacy services, care coordination, and all necessary supplies and equipment (drugs and nursing services coded separately), per diem / home therapy, hemodialysis; administrative services, professional pharmacy services, care coordination, and all necessary supplies and equipment (drugs and nursing services coded separately), per diem |

AV, arteriovenous; CM, clinical modification; CPT, Current Procedural Terminology; ESKD, end-stage kidney disease; ESRD, end-stage renal disease; ICD, International Classification of Diseases; i.v., intravenous; URR, urea reduction ratio

**Supplementary Table 3** Baseline comorbidities and medications

|  | **Moderate-to-severe  non-diabetic CKD (main cohort) *N* = 504,924** | **Moderate-to-severe  non-diabetic CKD with hypertension subgroup *N* = 428,867** | **Moderate-to-severe  non-diabetic CKD with hypertension and CAD subgroup N=113,239** |
| --- | --- | --- | --- |
| **Baseline comorbidities** | | | |
| Charlson-Deyo Comorbidity Index, mean ± SD | 3.4 ± 1.7 | 3.5 ± 1.7 | 4.3±1.9 |
| Charlson-Deyo Comorbidity Index, *n* (%) |  |  |  |
| 2 | 191,763 (38.0) | 156,121 (36.4) | 17,164 (15.2) |
| 3 | 120,521 (23.9) | 103,933 (24.2) | 24,960 (22.0) |
| 4 | 90,538 (17.9) | 78,344 (18.3) | 26,149 (23.1) |
| 5 | 50,184 (9.9) | 44,731 (10.4) | 20,357 (18.0) |
| ≥ 6 | 51,918 (10.3) | 45,738 (10.7) | 24,609 (21.7) |
| Hypertension | 428,867 (84.9) | 428,867 (100.0) | 113,239 (100.0) |
| Hyperlipidemia | 344,610 (68.2) | 305,064 (71.1) | 94,058 (83.1) |
| Hypothyroidism | 129,616 (25.7) | 109,856 (25.6) | 29,974 (26.5) |
| Anemia | 127,423 (25.2) | 114,163 (26.6) | 38,542 (34.0) |
| Pulmonary disease | 122,012 (24.2) | 106,247 (24.8) | 37,788 (33.4) |
| CAD | 121,368 (24.0) | 113,239 (26.4) | 113,239 (100.0) |
| Heart failure | 79,935 (15.8) | 74,356 (17.3) | 42,933 (37.9) |
| Depression and other mood affective disorders | 78,353 (15.5) | 65,391 (15.2) | 18,519 (16.4) |
| Atrial fibrillation | 75,294 (14.9) | 68,977 (16.1) | 33,357 (29.5) |
| Vascular disease | 71,157 (14.1) | 65,199 (15.2) | 28,163 (24.9) |
| Peripheral arterial disease | 69,661 (13.8) | 63,487 (14.8) | 28,260 (25.0) |
| Edema | 66,890 (13.2) | 61,296 (14.3) | 21,127 (18.7) |
| Obesity | 59,650 (11.8) | 54,410 (12.7) | 14,063 (12.4) |
| Transient ischemic attack | 35,973 (7.1) | 33,540 (7.8) | 14,299 (12.6) |
| Myocardial infarction | 31,691 (6.3) | 29,594 (6.9) | 29,554 (26.1) |
| Hypokalemia | 24,059 (4.8) | 22,373 (5.2) | 7496 (6.6) |
| Angina | 20,042 (4.0) | 18,779 (4.4) | 18,779 (16.6) |
| Hyperkalemia | 16,931 (3.4) | 15,650 (3.6) | 5411 (4.8) |
| Ischemic stroke | 16,485 (3.3) | 15,480 (3.6) | 6565 (5.8) |
| **Specific CKD diagnoses** |  |  |  |
| Tubulo-interstitial kidney disease | 64,135 (12.7) | 56,439 (13.2) | 17,578 (15.5) |
| Glomerulonephritis (chronic or unspecified) | 24,728 (4.9) | 21,213 (4.9) | 5856 (5.2) |
| Polycystic kidney disease | 8299 (1.6) | 7529 (1.8) | 1860 (1.6) |
| Acute glomerulonephritis | 539 (0.1) | 482 (0.1) | 82 (0.1) |
| IgA nephropathy (Berger’s disease) | 423 (0.1) | 367 (0.1) | 47 (<0.1) |
| **Baseline medication use** | | | |
| Centrally acting hypertensive | 361,000 (71.5) | 334,462 (78.0) | 90,747 (80.1) |
| Statin | 239,015 (47.3) | 211,908 (49.4) | 71,957 (63.5) |
| Beta-blocker | 222,183 (44.0) | 205,707 (48.0) | 72,985 (64.5) |
| NSAIDs | 179,762 (35.6) | 156,932 (36.6) | 38,782 (34.2) |
| ACE inhibitor | 174,059 (34.5) | 164,934 (38.5) | 43,212 (38.2) |
| Antidepressant | 169,682 (33.6) | 147,853 (34.5) | 38,135 (33.7) |
| Thiazide diuretics | 117,301 (23.2) | 112,659 (26.3) | 23,726 (21.0) |
| Calcium channel blockers | 108,535 (21.5) | 106,238 (24.8) | 27,353 (24.2) |
| ARB therapy | 103,871 (20.6) | 100,732 (23.5) | 25,876 (22.9) |
| Loop-acting diuretics | 83,584 (16.6) | 76,820 (17.9) | 32,859 (29.0) |
| Alpha-blocker | 67,823 (13.4) | 58,579 (13.7) | 20,070 (17.7) |
| Potassium supplement | 55,724 (11.0) | 51,279 (12.0) | 18,940 (16.7) |
| Oral anticoagulant | 49,817 (9.9) | 44,744 (10.4) | 19,273 (17.0) |
| Epithelial sodium channel blocker | 45,651 (9.0) | 41,717 (9.7) | 8896 (7.9) |
| Antiplatelet therapy | 36,550 (7.2) | 34,271 (8.0) | 23,702 (20.9) |
| Potassium-sparing diuretics | 27,516 (5.4) | 25,176 (5.9) | 8812 (7.8) |
| Spironolactone | 21,054 (4.2) | 19,102 (4.5) | 7759 (6.9) |
| Antiarrhythmics | 15,525 (3.1) | 14,218 (3.3) | 7978 (7.0) |
| Potassium-binding agents | 1786 (0.4) | 1652 (0.4) | 550 (0.5) |
| Eplerenone | 649 (0.1) | 604 (0.1) | 307 (0.3) |

ACE, angiotensin-converting enzyme; ARB, angiotensin receptor blocker; CAD, coronary artery disease; CKD, chronic kidney disease; IgA, immunoglobulin A; NSAID, nonsteroidal anti-inflammatory drug; SD, standard deviation

**Supplementary Table 4** Baseline demographics and laboratory values for patients with moderate-to-severe non-diabetic CKD and hypertension, with or without CAD

|  | **Moderate-to-severe  non-diabetic CKD and hypertension, without CAD* *N* = 428,867** | **Moderate-to-severe  non-diabetic CKD and hypertension, with CAD^†^ *N* = 113,239** |
| --- | --- | --- |
| Age, years, mean ± SD | 74.0 ± 10.1 | 76.7 ± 8.2 |
| Gender, *n* (%) |  |  |
| Female | 258,858 (60.4) | 54,073 (47.8) |
| Missing | 59 (< 0.1) | 14 (< 0.1) |
| Race, *n* (%) |  |  |
| White | 266,635 (62.2) | 74,342 (65.7) |
| Black | 43,818 (10.2) | 9586 (8.5) |
| Unknown | 35,417 (8.3) | 9449 (8.3) |
| Hispanic | 30,260 (7.1) | 7432 (6.6) |
| Asian | 8029 (1.9) | 1699 (1.5) |
| Missing | 44,708 (10.4) | 10,731 (9.5) |
| Index CKD stage CKD 3  CKD 4 | 404,516 (94.3)  24,351 (5.7) | 105,657 (93.3)  7582 (6.7) |
| Baseline eGFR, mL/min/1.73 m^2^,  mean ± SD | 51.3 ± 9.8  (*n* = 260,232) | 50.7 ± 10.1  (*n* = 66,412) |
| Baseline UACR, mg/g, median (IQR) | 10.0 (3.0–35.0)  (*n* = 25,913) | 12.0 (4.0–43.0)  (*n* = 5,990) |
| Baseline UACR, mg/g, *n/N* (%) |  |  |
| < 30 | 18,743 (4.4) | 4126 (3.6) |
| 30–≤ 300 | 5619 (1.3) | 1501 (1.3) |
| > 300 | 1551 (0.4) | 363 (0.3) |
| Missing | 402,954 (94.0) | 107,249 (94.7) |
| Serum potassium, mmol/l, mean ± SD | 4.0 ± 0.5 (*n* = 264,206) | 4.0 ± 0.5  (*n* = 67,676) |

*Patients were required to have at least 1 inpatient or 2 outpatient (on 2 different days) diagnosis codes for hypertension in the baseline period

**^†^**Patients were required to have at least 1 inpatient or 2 outpatient (on 2 different days) diagnosis codes for hypertension and for CAD in the baseline period

CAD, coronary artery disease; CKD, chronic kidney disease; eGFR, estimated glomerular filtration rate; IQR, interquartile range; SD, standard deviation; UACR, urine albumin-to-creatinine ratio

**Supplementary Table 5** Top 20 specialties of providers providing index diagnosis for CKD stage 3 or 4.

| **Specialty** | **n (%)** |
| --- | --- |
| Internal medicine | 52,060 (18.50) |
| Family medicine | 50,703 (18.02) |
| Clinical medical laboratory | 22,485 (7.99) |
| Nephrology | 19,689 (7.00) |
| General acute care hospital | 6403 (2.28) |
| Specialist | 3515 (1.25) |
| Cardiovascular disease | 2680 (0.95) |
| Hematology and oncology | 2101 (0.75) |
| Geriatric medicine | 1953 (0.69) |
| Home health | 1796 (0.64) |
| Epilepsy unit | 1491 (0.53) |
| Emergency medicine | 1490 (0.53) |
| Family | 1352 (0.48) |
| General practice | 1198 (0.43) |
| Nurse practitioner | 1190 (0.42) |
| Health maintenance organization | 1181 (0.42) |
| Student in an organized health care education or training program | 1148 (0.41) |
| Pathology | 1076 (0.38) |
| Physician assistant | 1054 (0.37) |
| Rehabilitation unit | 1038 (0.37) |
| **Missing** | **64,306 (22.86)** |

Provider specialties retrieved from unique provider IDs within Optum Clinformatics^®^ Data Mart database. Total number of patients indexed with CKD stage 3 or 4 was 281,350.

CKD, chronic kidney disease

**Supplementary Table 6** Outcomes in patients with moderate-to-severe non‑diabetic CKD with hypertension and CAD

|  | ***n*/total (%)** | **Incidence rate *n*/100 PY*** |
| --- | --- | --- |
| **Primary outcomes** |  |  |
| Hospitalization for HF | 22,928/113,185 (20.3) | 8.7 |
| ESKD/kidney failure/need for dialysis | 35,932/113,185 (31.8) | 15.7 |
| ESKD/need for dialysis | 6660/113,185 (5.9) | 2.4 |
| Kidney failure (acute and unspecified) | 34,290/113,185 (30.3) | 14.7 |
| Need for dialysis | 1828/113,073 (1.6) | 0.6 |
| CKD category worsening | 16,125/113,185 (14.3) | 6.2 |
| **Secondary outcomes** |  |  |
| **Cardiovascular** |  |  |
| Hospitalization for incident HF | 6450/61,560 (10.5) | 4.0 |
| Stroke | 15,385/113,185 (13.6) | 5.8 |
| Myocardial infarction | 35,629/113,185 (31.5) | 17.1 |
| Atrial fibrillation (new onset) | 13,170/75,690 (17.4) | 7.3 |
| **Kidney** |  |  |
| Acute kidney injury | 32,183/113,185 (28.4) | 13.4 |
| Kidney transplant | 453/113,125 (0.4) | 0.2 |
| eGFR decrease ≥ 30% | 4144/62,398 (6.6) | 2.7 |
| eGFR decrease ≥ 40% | 2058/62,398 (3.3) | 1.3 |
| eGFR decrease ≥ 57% | 571/62,135 (0.9) | 0.4 |

*Incidence rate calculated with the Aalen–Johansen estimator

CAD, coronary artery disease; CKD, chronic kidney disease; eGFR, estimated glomerular filtration rate; ESKD, end-stage kidney disease; HF, heart failure; PY, patient-years

**Supplementary Table 7** Time-course analysis of eGFR values over a 2-year period

| **Time** | ***n**** | **Relative frequency^†^ (%)** | **Mean eGFR (±SD)** |
| --- | --- | --- | --- |
| Index | 295,174 | 58.49 | 49.63 (± 8.36) |
| Y1 Q1 | 295,231 | 58.50 | 50.40 (± 9.27) |
| Y1 Q2 | 280,101 | 55.50 | 50.90 (± 10.01) |
| Y1 Q3 | 263,404 | 52.19 | 51.25 (± 10.53) |
| Y1 Q4 | 240,434 | 47.64 | 51.56 (± 10.99) |
| Y2 Q1 | 213,637 | 42.33 | 51.98 (± 11.43) |
| Y2 Q2 | 199,141 | 39.46 | 52.15 (± 11.80) |
| Y2 Q3 | 183,953 | 36.45 | 52.36 (± 12.15) |
| Y2 Q4 | 168,729 | 33.43 | 52.65 (± 12.52) |

*Patients with at least one eGFR measurement, LOCF, or imputation available for a given time period

^†^Proportion of patients included in this analysis out of the total number of patients with follow-up data available

eGFR, estimated glomerular filtration rate; LOCF, last observation carried forward; Q, quarter; SD, standard deviation; Y, year

**Supplementary Table 8** eGFR and albuminuria categories, and CKD stages*

| **eGFR Category** | **Description** | **eGFR, mL/min/1.73 m^2^** |
| --- | --- | --- |
| G1 | Normal or high | ≥ 90 |
| G2 | Mildly decreased^†^ | 60–89 |
| G3a | Mildly to moderately decreased | 45–59 |
| G3b | Moderately to severely decreased | 30–44 |
| G4 | Severely decreased | 15–29 |
| G5 | Kidney failure | 15 |
| **Albuminuria category** |  | **UACR (mg/g)** |
| A1 | Normal to mildly increased | < 30 |
| A2 | Moderately increased^†^ | 30–300 |
| A3 | Severely increased | > 300 |
| **CKD stage** | **Description** | **Criteria** |
| 1 | Kidney damage with normal GFR | GFR category G1 and albuminuria category A2 or A3 |
| 2 | Kidney damage with mildly decreased GFR | GFR category G2 and albuminuria category A2 or A3 |
| 3a^#^ | Mildly to moderately decreased GFR | GFR category G3a |
| 3b^#^ | Moderately to severely decreased GFR | GFR category G3b |
| 4 | Severely decreased GFR | GFR category G4 |
| 5 | Kidney failure | GFR category G5 |

*Based on KDOQI 2002 guidelines

^†^Relative to young adult level

^#^In the current study, patients with CKD stages 3a and 3b were categorized into a single stage (CKD stage 3)

CKD, chronic kidney disease; G, grade; GFR, glomerular filtration rate; KDOQI, Kidney Disease Outcomes Quality Initiative; UACR, urine albumin-to-creatinine ratio


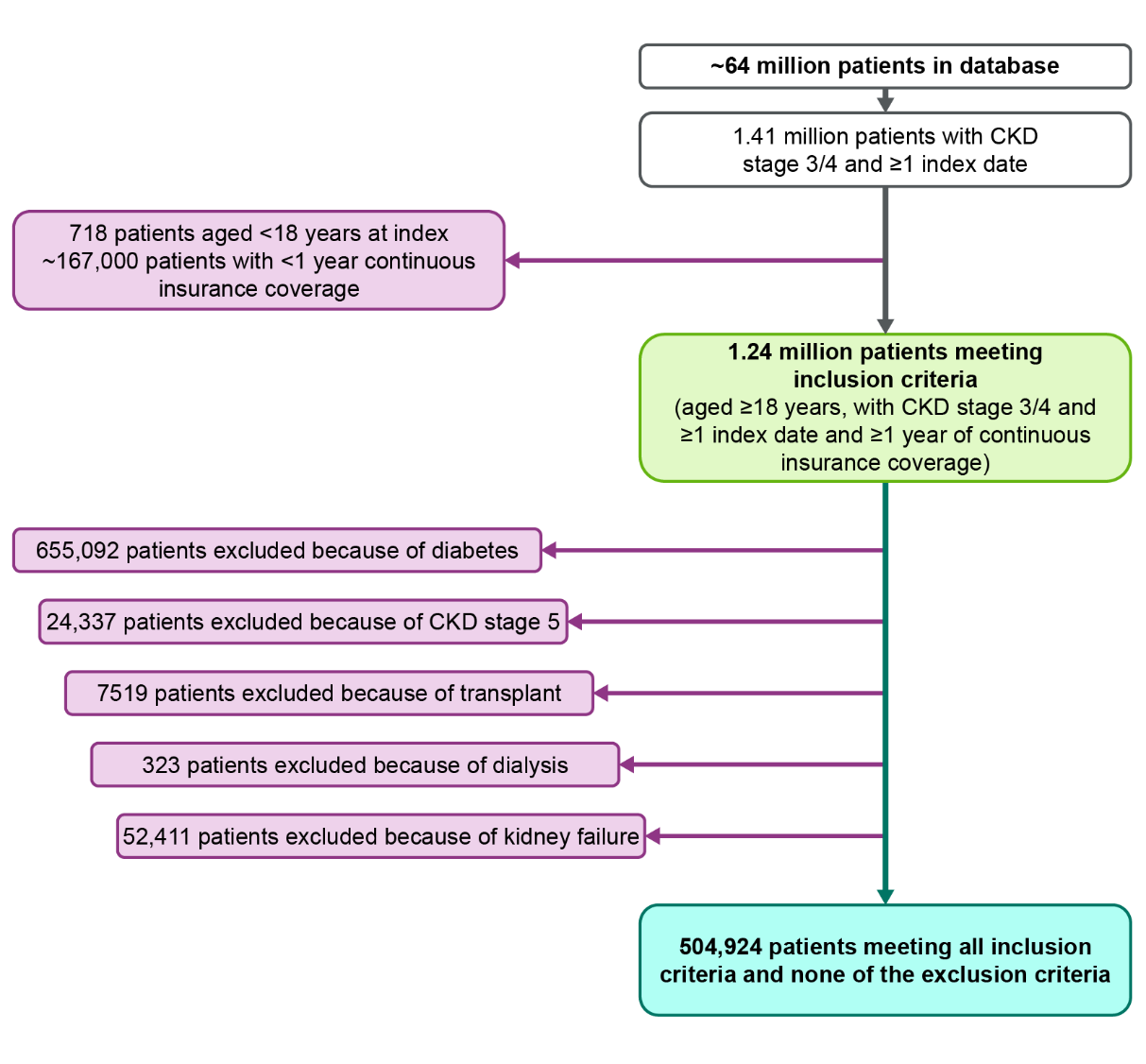


**Supplementary Figure 1** Patient flowchart

CKD, chronic kidney disease.


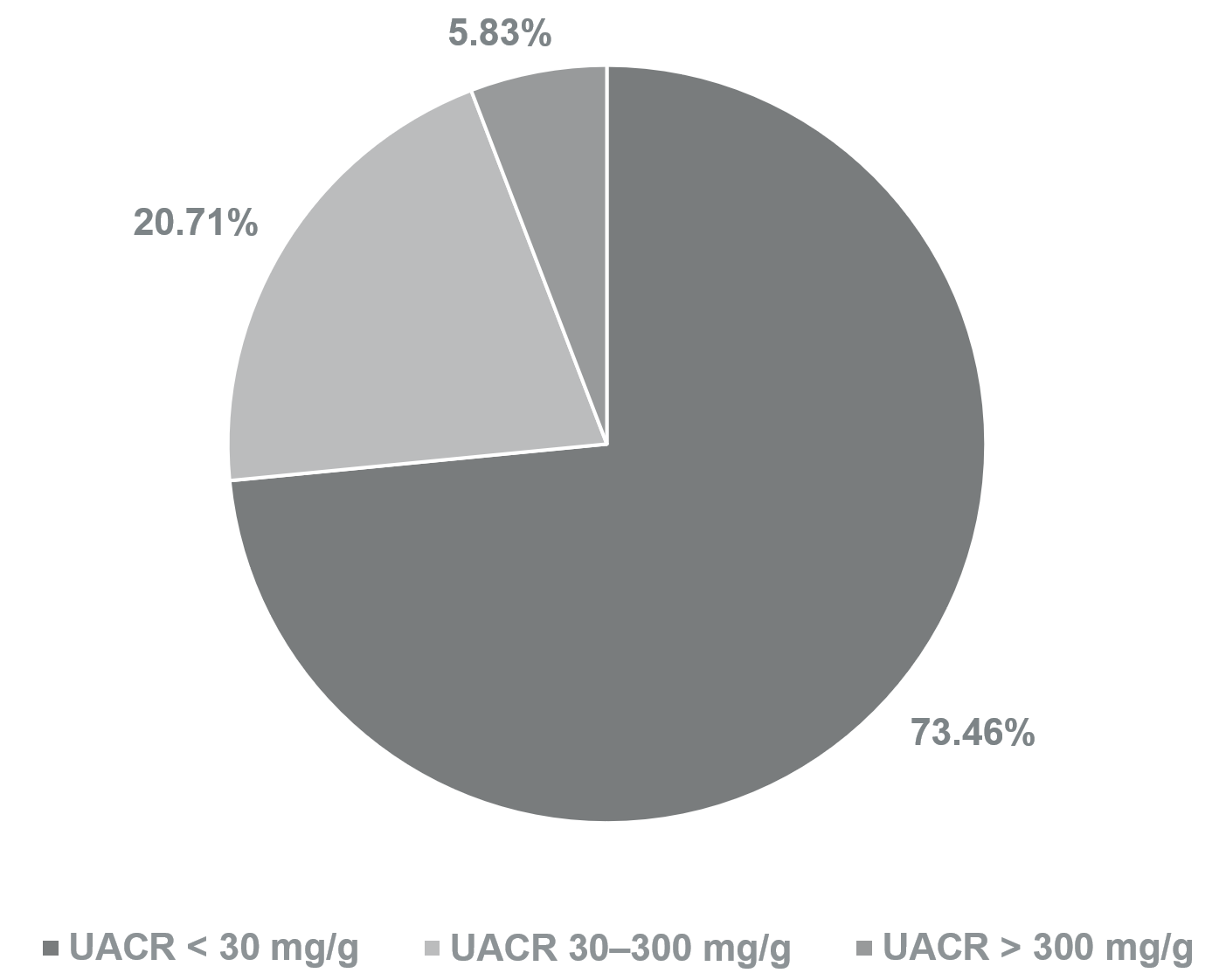


**Supplementary Figure 2** Baseline UACR distribution in patients with available UACR measurements (*n* = 30,793)

UACR, urine albumin-to-creatinine ratio.


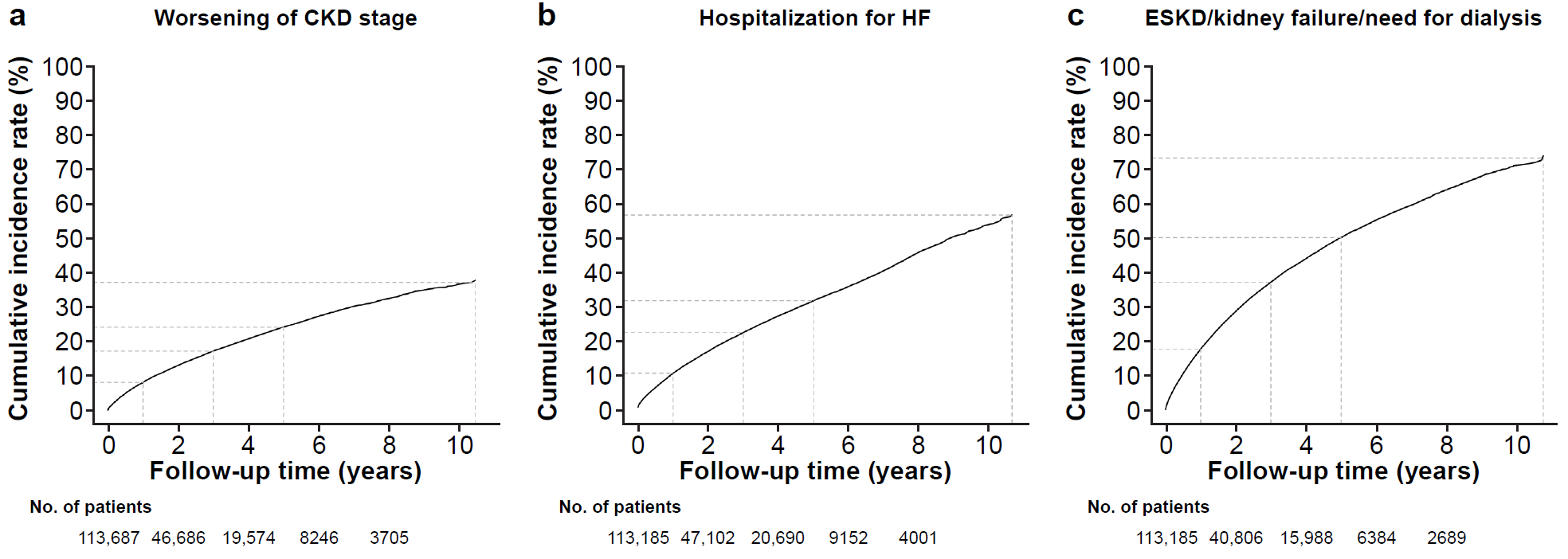


**Supplementary Figure 3** Aalen–Johansen cumulative incidence rate curves for primary outcomes in patients with moderate-to-severe non-diabetic CKD with hypertension and CAD. Worsening of CKD stage from baseline (**a**) HF hospitalization and (**b**) ESKD/kidney failure/need for dialysis (**c**). Dashed lines show the survival rate at specified time points

CAD, coronary artery disease; CKD, chronic kidney disease; ESKD, end-stage kidney disease; HF, heart failure


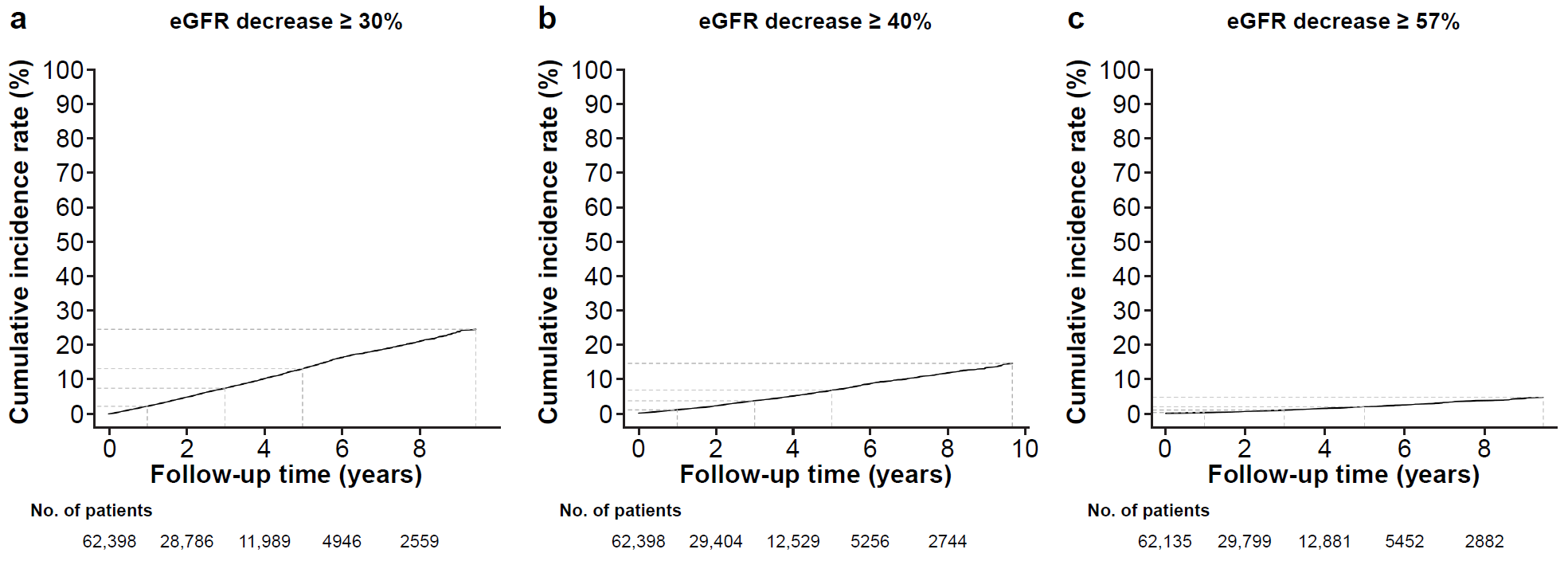


**Supplementary Figure 4** Aalen–Johansen cumulative incidence rate curves for categorical eGFR decreases in patients with moderate-to-severe non-diabetic CKD with hypertension and CAD. Decrease in eGFR of ≥ 30% **(a)**, ≥ 40% **(b)**, and ≥ 57% **(c)**, assessed using actual measured values with no imputation. Horizontal dashed lines show the survival rate at specified time points

CAD, coronary artery disease; CKD, chronic kidney disease; eGFR, estimated glomerular filtration rate


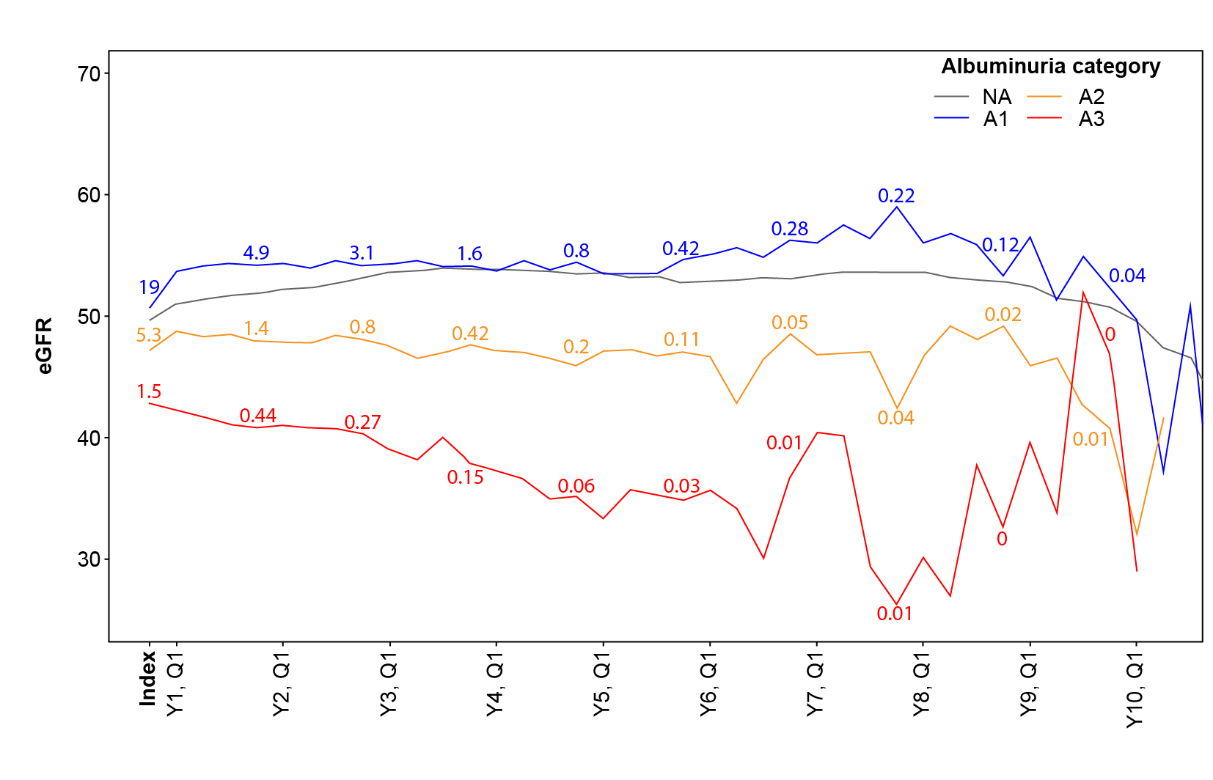


**Supplementary Figure 5** eGFR over time by albuminuria category

Albuminuria categories were as follows: A1: UACR < 30 mg/g; A2: 30–300 mg/g; A3: > 300 mg/g. Numbers on the graph show the number of patients (1000s) for each albuminuria category at the respective timepoints

eGFR, estimated glomerular filtration rate; NA, not available; Q, quarter; UACR, urine albumin-to-creatinine ratio; Y, year


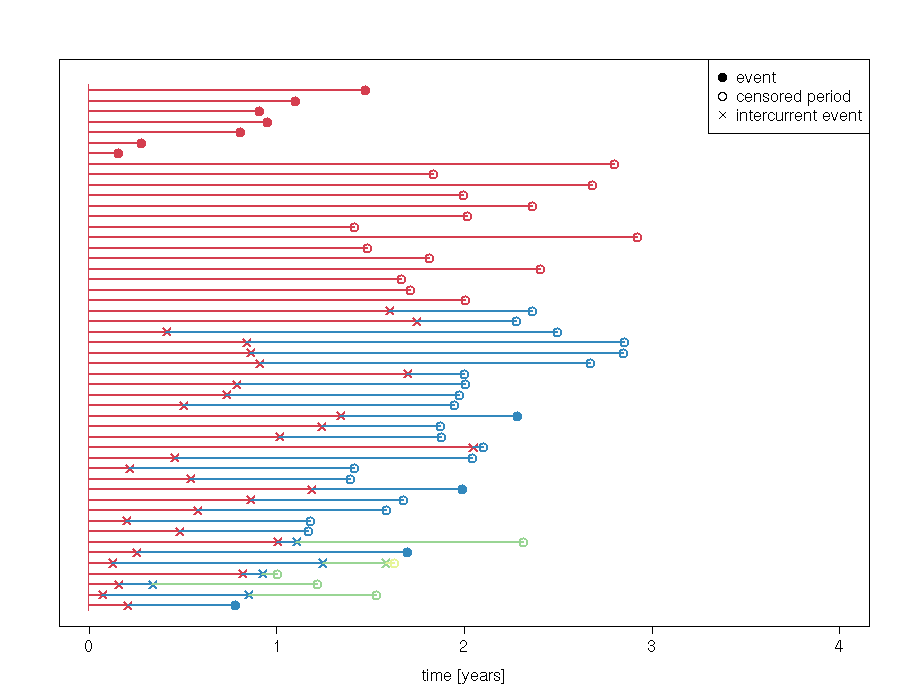


**Supplementary Figure 6** Illustrative figure depicting a scheme of time-to-event data including intercurrent events. Each line represents one patient. The follow-up period of a patient begins at 0 and ends with an open or a filled circle. The filled circle indicates an event (e.g., HHF), the open circle indicates the end of the patient follow-up period (right-censored period). Intercurrent events are indicated with “x”

HHF, hospitalization for heart failure
